# Students' experience of being seen by their physical education teachers and associated factors

**DOI:** 10.3389/fspor.2023.1101072

**Published:** 2023-04-17

**Authors:** Fredrik Andresen, Eli Torvik, Pål Lagestad

**Affiliations:** ^1^ Physical Education and Sports Science, Nord University, Levanger, Norway; ^2^ Institute of Teacher Education, Norwegian University of Science and Technology, Trondheim, Norway

**Keywords:** physical education, being seen, high school, students, teacher

## Abstract

**Background:**

The experience of being seen by physical education (PE) teachers is an important pedagogical term in school settings, and is closely related to the theory of recognition pedagogy and self-determination theory. However, very few studies have been conducted concerning this term, and extant research has typically been based on small sample sizes, and thus is unlikely to be extrapolated to other contexts.

**Purpose:**

The aim of the study was to examine the extent to which students experience being seen by their PE teachers, which factors constitute the phenomenon of being seen as a pedagogical term, and how these factors correlate with students' experience of being seen by their PE teachers. This is the first study to identify factors that constitute the pedagogical term *being seen*, and uses a quantitative design.

**Method:**

A questionnaire was developed on the basis of theory and previous research, and data from 412 students were collected. Principal component analysis was conducted to examine the dimensionality of the questions and which factors could be associated with *being seen*. From this, indexes were subsequently created for each factor. The association between these factors and the experience of being seen was determined using Spearman's correlation test.

**Results:**

The results showed that 76.2% of the students reported being seen by the teacher in PE, while 7.8% reported not being seen, and 16.1% of the students neither disagreed nor agreed to being seen by the teacher in PE. The factor analysis indicated that being seen may be related to students' experience of the following: being able to display their skills; the teacher's caring behavior; feedback from the teacher; dialogue with the teacher; and evaluation and goals. The correlation analysis showed that these five factors correlated significantly at a medium level with the students' experience of being seen by their PE teacher.

**Conclusion:**

The results point toward the importance of PE teachers giving their students opportunities to display their skills, providing the students with feedback through good dialogue, showing them that their teachers care, and involving students in evaluation and establishing goals in PE.

## Introduction

It is well demonstrated that it is critical for students that their basic social and psychological needs be met in order to enjoy and master their schooling. To a considerable degree, *being seen* is linked to this through recognition, a sense of belonging, and support (1, 2). In Norway, the pedagogical term used by many teachers related to this process is *being seen*. The importance of being seen expresses what physical education (PE) teachers (and other teachers) say in everyday terms related to students' recognition, sense of belonging, and support by the teachers. Skrøvset, Mausethagen and Slettbakk (3) pointed out that to be validated about whom we are and what we do is essential, and that it is difficult for students to express themselves authentically without being validated by their teacher. In addition, Vedul-Kjelsås and Elnan (4) emphasized the importance of teachers being in close dialogue with students, and facilitating good learning experiences by asking them about their needs and interests, and making PE lessons as beneficial and enjoyable as possible for every student. Being supported by the teacher relates to the feeling of being seen, and it is crucial that the teacher displays an interest in the students' lives and gives them the feeling of being seen (5, 6). For this reason, it is worthwhile to examine students’ experience of being seen in physical education (PE), and which factors that relate to the pedagogical term *being seen* in PE in Norway.

A comprehensive literature search indicted that what constitutes the term *being seen* and which factors relate to the pedagogical term *being seen* is a research area with a paucity of pertinent literature. By using the terms “being seen” and “school”, and “teacher support” and “school”, the literature search identified 1,923 articles. By reading the titles, then the abstracts, and finally the text, 17 articles related to being seen were identified (1, 6–11). Most of these studies are indirectly associated with the pedagogical term *being seen*. Sparks et al. (8, 9) showed how belonging-supportive teachers notice and recognise emotions and events among students. Both Smith and St. Pierre (12) and Whittle et al. (10, 11) highlighted the importance of the teacher's ability to communicate and convey knowledge clearly as determinative for students' feeling.

However, only two studies from Norway directly addressed the concept of *being seen* by PE teachers (1, 7). Both investigations drew on data from interviews with students. Author (b) (1) found that students at upper secondary school experienced themselves as being seen in either a positive or negative manner. The findings of this study also showed that this feeling of being seen was relatively stable among students over time. In their study, Author (a) (7) examined what constitutes being seen from a student's perspective. A central result in this study was that the experience of being seen concerned being able to display one's abilities, feeling that the teacher cares, the quality and tone of the dialogue with the teacher, and the teacher's feedback.

Theoretically, it can be argued that the key for PE teachers in making provision for these factors in school lies in what Jordet (2) terms *recognition pedagogy*. Jordet (2) refers to “recognition” as deriving from the German word “*annerkennen*”, which means to give praise, respect, or appreciate. It constitutes acknowledging that what a person says, does, or is, is sufficient (2). This pedagogy addresses the three forms of recognition described by Honneth (13), but places them in a pedagogical context that the teacher can work with in school. Specifically, this comprises rights in the public sphere, love in the private sphere, and valuing in the social sphere.

Rights in the public sphere refers to the principle that each person is of equal value, irrespective of social status, gender, ethnicity, religion, ability, etc (13). From a school perspective, it means that children experience active participation in the classroom community and are viewed as equal partners in these interactions. This is achieved by adapting the education content to each individual, supporting students to make the best use of their resources, and creating a learning environment with the requisite resources for the students to develop and attain satisfactory learning outcomes. If the education is successful with these pedagogical approaches, the students will develop an appreciation of their inherent worth and right to freedom, and will be helped in developing self-esteem (2, 13).

Jordet (2) suggested that expressing love is both a relevant and central aspect of the teaching profession. To experience love through care, empathy, and warmth in social relations is a fundamental need, and should be included in all teachers' meetings with students (2). “Agape” and “storge” are forms of other-oriented love, which Jordet (2) believes describe what love in the teaching profession entails. “Agape” is a concept of love within Christianity, and indicates unconditional and selfless love of the other based on respect. The concept includes that this display of love derives from making the needs of the other central. “Storge” constitutes a form of love in which the person, in this case the teacher, exhibits care, empathy, and warmth toward the student; this can be closely linked to the teacher's mandate and educational role by also focusing on motivating, correcting, and challenging the student.

Social validation, as a form of recognition, arises from the human need to feel that one is contributing one's resources and being valued by those around one for this contribution (13). It is well-known that students tend to differ in various aspects, and come to school from markedly dissimilar backgrounds. This represents a challenge if the school is to value all of the different competencies and resources found in every single class in Norwegian schools. Adapted education is, however, mandated in Norwegian schools, and thus it is crucial that the schools identify systems and tools that value the diverse achievements of a complex group of students from widely different backgrounds. Students develop and reinforce their self-confidence through experiencing social validation, and overcoming the academic and social challenges that they encounter at school.

Self-determination theory emphasizes the importance of the social environment of students for personal growth (14). Deci and Ryan (14) identified three basic needs in all humans: autonomy; relatedness; and competence. These factors are essential for optimal motivation, integration, and well-being, which in turn lead to intrinsic motivation. Indeed, intrinsic motivation is the prototype of self-determination in their self- determination theory. A teacher can encourage or impede students' intrinsic motivation by taking these three basic needs into consideration, and in terms of *being seen*, relatedness seems highly important. Relatedness concerns feeling cared for and connected to someone (14). Deci and Ryan (14) claimed that students need to feel connected with others, i.e., to care and be cared for (the need for relatedness).

As pointed out earlier, only two studies have directly examined the importance of being seen in PE. These studies (1, 7) are both qualitative studies with relatively few participants; although they provide rich, in-depth insight, they are not generalizable. The findings of these investigations and others that focus on recognition and belonging in school identify certain overarching factors that lead to the student being seen by his or her teacher. It will, therefore, be worthwhile to elucidate these factors in a more generalisable way. It will also be important, with the assistance of these factors, to develop a questionnaire which can be used to capture the phenomenon of *being seen* in physical education at school. Using a quantitative design, the aim of this study was to examine the extent to which students' experience being seen by their teachers in PE, which factors constitute the phenomenon of being seen, and how do these factors correlate with students experience of being seen.

## Method

To achieve the purpose of the study, a questionnaire was developed, and factor analysis and correlation analysis were used to identify significant factors related to being seen. Ethical standards were followed in accordance with the ethical guidelines of the Norwegian National Research Ethics Committees (15) and the Declaration of Helsinki. Approval to use the research data and conduct the study was granted by the Norwegian Centre for Research Data (NSD).

### Participants

With the assistance of a stratified selection intended to form a representative group (16), two medium-sized high schools (aged 16–19 years-old) in mid-Norway were chosen for the study. These schools had between 5 and 10 physical education (PE) teachers each. The schools themselves decided which classes would take part in the study. The study included 412 students aged 16–19 (208 boys, 200 girls, and four students did not report their gender), from different study programmes, who gave valid answers to the questionnaire. The gender and study programme distribution, presented in Table 1, reflect the natural distribution across Norwegian upper secondary schools.

**Table 1:** Descriptive data of study programme and year group (first, second, and third year at high school).

Study programme	VG1 (*N*)	VG2 (*N*)	VG3 (*N*)	Total (*N*)
Art and design	10	9	4	23
Media and communication	20	9	2	31
Specialisation in general studies	77	69	48	194
Building and construction		18		18
Electrical and computer technology	34	14		48
Information technology and media production		5		5
Restaurant and cookery	20	9		29
Sales, service, and public transport	10			10
Technology and industry	8	12		20
Health and child development	17	17		34
Total	196	162	54	412

### Procedure

The initial phase of developing the questionnaire involved carrying out a thorough review of theory and research in the field, in order to be fully informed about the topic and to ensure that the questions were in line with earlier literature (16, 17). With a strategy of using previous validated questionnaires, the intention was to develop the concept of *being seen* using previous theory and research within an explorative design. The two studies related to the pedagogical term *being seen* were included, as well as other studies that focused on students' recognition, sense of belonging, and support by teachers. Procedurally, this adaption of the questionnaire was in accordance with those produced by Beauchamp et al. (18), Säfvenbom, Buch and Aandstad (19), and Sparks et al. (17). Respectively, they aimed at designing and validating TTQ (Transformation Teaching Questionnaire), the EPAS (Eagerness for Physical Activity Scale), and a model for students' experience of being supported in belonging by their PE teacher. Many of the questions drew on those used previously to elucidate relevant topics, but some questions were formulated ourselves. In reviewing theory and earlier literature around these research questions, four areas appeared to be central to the experience of being seen: (1) the importance that the teacher cared; (2) feedback from the teacher; (3) that the student could show what he or she could do; and (4) good dialogue with the teacher. The questionnaire comprised 51 questions. A seven-point Likert-scale, running from left to right from most negative to most positive, respectively, was used (totally disagree, disagree, disagree a little, neither disagree/agree, agree a little, agree, totally agree).

Beauchamp et al.'s (18) Transformational Teaching Questionnaire (TTQ) and the questions (statements) “Shows that he or she cares about me”, “Tries to know every student in the class”, “Attempts to help students who might be struggling”, and “Recognizes the needs and abilities of each student in the class” were used in terms of caring (questions 13–14 and 17–20). Questions about feedback from the teacher, and their validated tools for measuring perceived verbal and non-verbal feedback from the teacher, were taken from Koka and Hein (20), and reworked. These questions were: “My work is frequently encouraged by the teacher”, “The teacher often praises me”, “When I do well in PE, the teacher confirms that”, and “The teacher often gives me instruction/the teacher instructs me frequently during the performance” (questions 21–25). Questions about being able to display one's skills were specially formulated, as they are absent from earlier research. Question 34 is taken from Beauchamp et al. (18) as: “Recognizes the needs and abilities of each student in the class”. Questions about good dialogue with the teacher were taken from Metheny, McWhirter and O'Neil (21), and reworked. Their study was based on McWhirter's (22) Teacher Assessment Scale (TSS). They used the following questions: “Most teachers in my school will listen if I want to talk about a school problem”, “Most teachers in my school are easy to talk to about school things”, and “Most teachers in my school are easy to talk to about things besides school”. These questions measure what Metheny and colleagues (21) called *availability*. The questions were modified for this study and formed questions 44–46. In addition, the question: “My teacher is friendly and approachable”, from Sparks et al.'s (17) study served as a basis for questions 47–49. The other questions were formulated especially for this study on the basis of extant theory and research. Examining the content of the questions in the questionnaire, suggests that that the questions face validity are high (23).

Once the questionnaire was fully developed, a pilot study was carried out in February 2021. This was done to identify possible difficulties, misunderstandings, or shortcomings in the questionnaire which was distributed among a class of first-year students (VG1, 16-year-olds) at upper secondary school and a class of second-year students (VG2,17-year-olds). Together, 39 students responded to the pilot (20 from VG1 and 19 from VG2). In the pilot study, the students were encouraged to comment on difficulties with the questionnaire, if any. Most students did not make any specific comments about the difficulties they faced with any particular questions. However, a small number of students suggested that comments relating to how long they were taught by their PE teacher and questions relating to exercise inside and outside of school, were unclear. These were rewritten with these comments in mind. The data collection was also conducted in February 2021.

### Analyses

The experience of being seen by their teacher in PE was based on the following question: “I experience being seen by the teacher in PE”, and descriptive data, mean, and standard deviation were used. Principal components analysis (PCA) was carried out to determine how many components (factors) constituted the phenomenon of being seen, using an explorative strategy. Field (24) asserted that a sample of over 300 represents a sound basis for such an analysis, and this requirement was met. Questions 13–51 were included in the analysis. Questions 1–12 were not included, however, because these were included to elicit background information and not intended to shed light on aspects of *being seen*. The PCA resulted in a correlation matrix showing the correlation between all of the questions included in the analysis. Field (24) emphasised the importance of studying the correlation matrix to identify questions with correlations less than 0.3, and assessing whether or not they should be excluded. The Kaiser-Meyer-Olkin test was used to determine whether the data collection and selection were sufficient. An oblique rotation method was employed to find a readily understood and clear structured factor solution (23, 25). The pattern matrix was also set against eigenvalue and share of explained variance. Eigenvalue reveals how much of the variance is explained by each individual factor in the questions (25). Kaiser's criterion of removing factors with an eigenvalue greater than 1 was used (24, 25). The result of the PCA showed that the correlation matrix was not an identity matrix, in that Bartlett's test of sphericity was significant (*p* < 0.001). In addition, KMO showed a value of 0.966, which is significantly higher than the cut-off value of 0.5 indicated by Field (24), and therefore the dataset was suitable for factor analysis.

Furthermore, the pattern matrix was studied to determine which questions gave loading to which factors. Questions that gave loading to several factors were excluded together with questions that failed to load any factor. The cut-off for factor loading was set at 0.4, in accordance with Ringdal (23). In line with Thomas et al. (26) and Ringdal (23), content validation of the various factors was carried out in the form of a subjective assessment of what the questions measured. After the pattern matrix was reviewed, and all of the different questions were either assigned to a factor or excluded, indexes for the various factors were created. The design of the indexes ensured that all of the scores for each individual question within a factor were summed and divided by the number of questions in that factor. A Kolmogorov-Smirnov test (27) was performed to test the normality of the indexes (27).

A Spearman's rho correlation analysis was conducted to clarify the relationship between the factors generated by the PCA and the students' experience of being seen. The strength of the correlations was based on Cohen and Holliday's (28) correlation strength (0–0.19 very weak, 0.2–0.39 weak, 0.4–0.69 moderate, 0.7–0.89 high, 0.9–1 extremely high). Cronbach's alpha was used to establish the reliability of the factors and the associated questions as identified by PCA (24). A Cronbach's alpha above 0.7 was considered satisfactory (23, 27). In addition, the corrected item, i.e., total correlation, should be above 0.3 (24).

## Results

### Students' experience of being seen by the teacher

**Figure 1:**
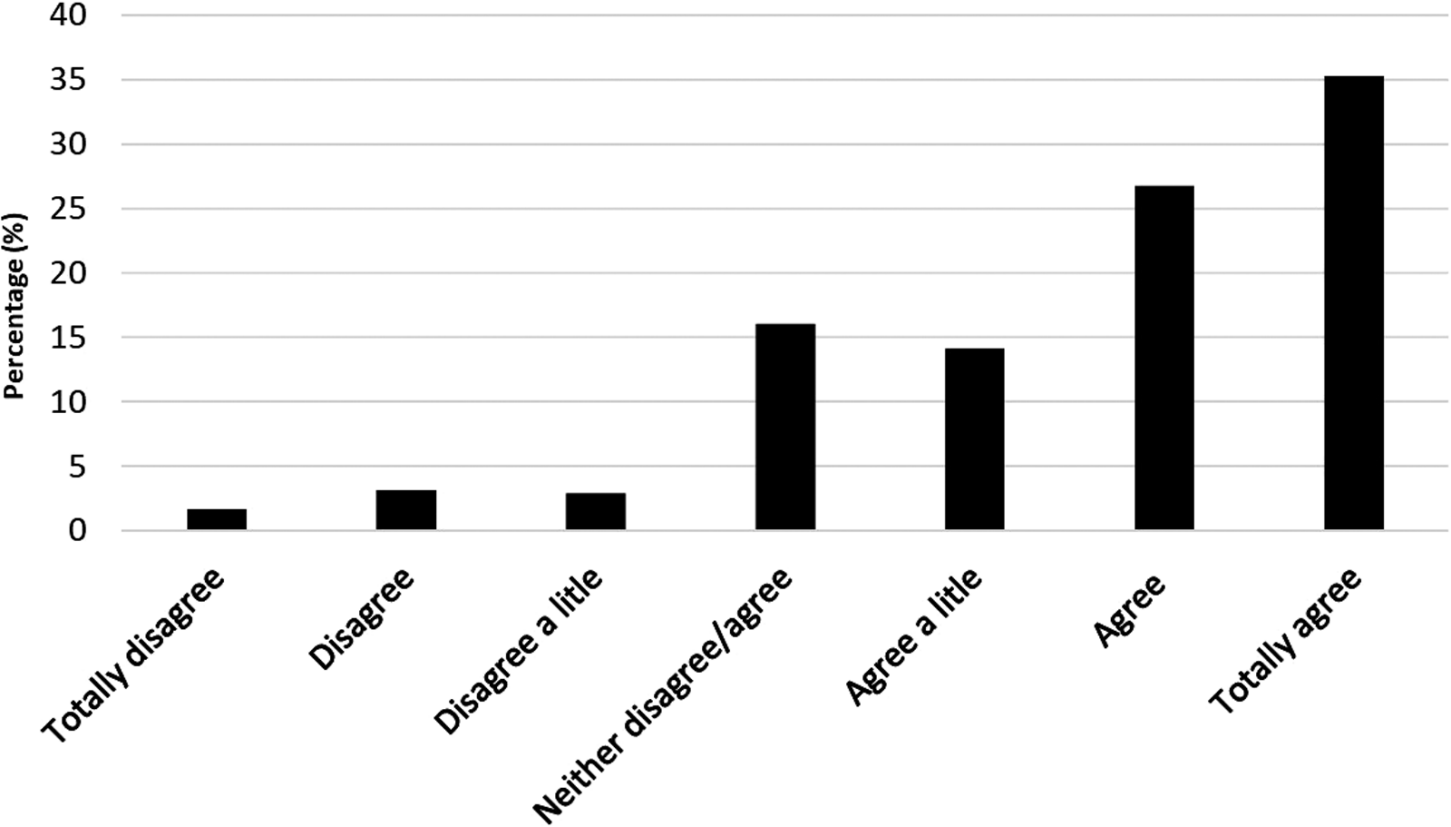
Distribution of answers to the statement: “I experience being seen by the teacher in PE.”

Figure 1 shows that 76.2% of the students, to varying degrees, experienced being seen by the teacher during PE. The data reveal, further, that, to varying degrees, 7.8% of the students experienced that they were not seen by the teacher in PE. Furthermore, 16.1% of the students neither disagreed nor agreed to being seen by the teacher in PE.

### Factors that constitute the experience of being seen

Table 2 shows the pattern matrix, and how the different questions fall into the various factors, and their factor loading.

**Table 2:** Pattern matrix with all of the questions in the questionnaire and their factor loading.

	Comp. 1	Comp. 2	Comp. 3	Comp. 4	Comp. 5
25 Gives me instructions during an activity	.792				
24 Gives me instructions before an activity	.780				
26 Tells me what to do well in PE	.675				
23 Gives me affirmation when I succeed in PE	.610				
22 Praises me in PE	.579				
27 Tells me what I can work on in PE	.547				
28 Motivates me	.539				
21 Encourages me in PE	.440				
39 Gives me manageable challenges					
42 Lets the most able share in decision-making in PE		.805			
40 Gives me challenges I can't manage		.660			
14 Cares about how things are for me outside PE			.913		
19 Notices my needs			.744		
20 Tries to get to know me			.740		
15 Notices if PE is not going well for me			.733		
17 Tries to help if he or she sees that I am struggling with something			.700		
13 Cares about how PE is going for me			.654		
16 Notices if there is something I can't do in PE			.615		
46 Is easy to speak about things outside of PE			.609		
18 Tries to help me if am struggling with something			.542		
49 Is easy to contact outside of PE			.492		
47 Is friendly				.829	
48 Is easy to make contact with in PE				.681	
45 Is easy to talk to about things in PE				.588	
51 Can explain in a way I understand				.565	
44 Listens if I need to talk about a problem				.505	
32 Lets me take part in my own evaluation					−.787
36 Asks me what I find difficult					−.778
37 Asks me what I find easy					−.726
38 Sets challenges well suited to me					−.566
35 Helps me to see what I'm good at					−.548
29 Sets targets for me in PE					−.519
30 Lets me set my own goals in the physical exercises					−.516
33 Gives me the opportunity to display my skills					−.498
34 Notices what I achieve	.403				−.447
31 Assesses me fairly					−.428
50 Has contact with me during the lesson					
41 Allows me to take part in decision-making in PE					
43 Arranges various activities so everyone can show their skills					

On the basis of the pattern matrix and previous research, four of the five components were given names. Component 1 (“Feedback”) included eight questions (Cronbach's alpha = 0.939). Component 2 was deleted because of its negative approach, and because it was difficult to interpret what phenomenon two such different questions measured. Component 3 (“teacher's caring”) comprised 10 questions (Cronbach's alpha = 0.946). Component 4 (“good dialogue”) included five questions (Cronbach's alpha = 0.910). Component 5 was divided into two factors. “Evaluation and goals” included four questions (questions 29–31, Cronbach's alpha = 0.871). “Display one's skills” included five questions (questions 33, 35–38, Cronbach's alpha = 0.915). Cronbach's alpha test was satisfactory for the five factors, correlating between 0.6 and 0.73 with the total score.

Table 3 presents descriptive statistics for the five indexes. The table shows that the students gave the highest average score to good dialogue; whereas, the lowest average score was given to displaying one's skills.

**Table 3:** Descriptive statistics for the five indexes.

Factor	*N*	Mean	Median	Standard deviation
Feedback	407	5.35	5.375	1.19
Teacher's caring	398	5.19	5.3	1.24
Good dialogue	407	5.85	6.0	1.14
Evaluation and goals	406	4.79	4.75	1.43
Display one's skills	403	4.57	4.6	1.38

### Relationship between the factors and the experience of being seen

The result of the correlation analysis in Table 4 reveals that all five factors forming the basis of being seen correlate significantly and positively with the experience of being seen. Although these correlations are moderate, some come close to being high correlations (28). There are also moderate-to-high correlations between all of the five factors constituting the phenomenon of being seen.

**Table 4:** Spearman's correlation test between the five factors and the experience of being seen.

	Experience of being seen	Feedback	Teacher's caring	Good dialogue	Evaluation and goals
Feedback	.655***				
Teacher's caring	.695***	.802***			
Good dialogue	.645***	.759***	.7.64***		
Evaluation and goals	.579***	.775***	.739***	.684***	
Display one's skills	.586***	.789***	.744***	.692***	.830***

*** Significant correlations at the .001 level.

## Discussion

### Students' experience of being seen by the teacher in PE

The results show that 76.2% of the students perceived themselves as being seen by the teacher during PE. However, the results also indicate that 7.8%, experienced not being seen by the teacher in PE. From a teacher's perspective, it can be argued that this is neither satisfactory nor optimal. According to Hattie (29), the teacher has a unique possibility of playing a critical role in the positive development of children and young people. Furthermore, Hattie points towards the teacher as the most critical factor related to student achievement in school about which improvements are highly feasible (29). Indeed, students themselves highlight the importance of the teacher's role in their school experience and academic achievement (10–12). On the other hand, the teachers' conditions which permit real observation of, and communication with, all of the students are often limited. Frequently, the classes consist of up to 28 students. As a consequence, being able to have good dialogue with every student, providing caring attention to every student, giving feedback to every student, including every student in evaluations, and arranging the PE class so that every student can display his or her skills is markedly challenging within the approximately 100 min per week when PE teachers meet the students. A research study (30) interviewing and observing PE teachers reports that it is much easier to support and provide feedback to students in a class with 15 students than in a class with 20 or more students.

### Factors which constitute the basis for being seen

The factor analysis and interpretation of it identified five factors that form the basis for students experiencing being seen by the teacher in PE. These five factors are: (1) feedback from the teacher; (2) feeling that the teacher cares; (3) having good dialogue with the teacher; (4) having evaluations and goal setting; and (5) being provided with opportunities to display his or her skills. Of these, feedback from the teacher, that the teacher cares, having good dialogue, and that the student has opportunities to display his or her abilities have been identified in previous literature (7) as being indicators of being seen. The findings from Author (a) (7) study were based on data collected from interviews. The current study, however, is based on factor analysis and quantitative data, which are generalisable to a greater extent. It is remarkable that the statistical factor analysis of the 39 questions corresponds so closely with the qualitative findings of Author (a) (7), and that four of the five factors correspond to the four factors highlighted in that study.

The first factor, caring, was also identified by Author (a) (7) in an interview study. The students in Author (a) (7) study indicated that being caring meant that the teacher showed interest in and care for them, and many of the questions in this factor can be linked to displaying interest and being caring. Ryan and Patrick (31) claimed that emotional support from the teacher means that the teacher exhibits understanding and kindness, and cares about the student, which can be associated with Jordet's (2) description of the form of recognition love, and the concepts of “agape” and “storge” (2). These concepts suggest that a person shows love by making the needs of the other central through empathy, warmth, and care. The teacher caring about the student largely concerns expressing interest, as well as facilitating their engagement and assisting them. Caring and showing love arise largely from what person the student is, rather than what that person does. Jordet (2) claims that love is a basic psychological need that must be attended to in all areas of life, including school. In addition, Deci and Ryan's (14) theory about relatedness as one of three basic needs in all humans is also essentially related to our finding about caring. Deci and Ryan (14) point to relatedness as feeling cared for and connected to someone, such as the PE teacher.

The importance of the teacher noticing and acknowledging aspects of the students is supported by findings of Sparks et al. (8), Sparks et al. (17), and Sparks et al. (9). They report that belonging-supportive teachers noticed and acknowledged emotions and events among students in the classroom. Students had a stronger belief that they can master their schoolwork and have higher professional and social competence if they experienced emotional support from their teacher (32, 33). This is in line with the relationship between teacher and student being one of the factors with the most powerful effect on students’ learning outcomes (34, 35). Findings from Smith and St. Pierre (12) also support the assertion that the teacher is significantly important for students’ experience of PE. As mentioned, people experience a sense of belonging when other people care about them.

The second factor, good dialogue, was also identified by Author (a) (7) in an interview study. Central aspects of the questions about this factor were: listening; being friendly; being easy to make contact with; and being a good communicator. That good dialogue can affect the experience of being seen is supported, directly or indirectly, by several researchers. Author (b) (1) found that students stated that the characteristics of being seen in a positive way were reflected, among other things, in good communication with the teacher, a result that is consistent with Smith and St. Pierre (12). Whittle et al. (10) and Whittle et al. (11) also identified the importance of the teacher's ability to communicate and to convey knowledge clearly. This implies not only that the teacher is adept at communicating with the students, but also that he or she is able to tailor the communication to the student's particular understanding and to be readily available for questions and interactions. The teacher being available was described as the teacher being easy to get in touch with and taking the time to see and assist the students. The importance of good dialogue is supported by Baumeister and Leary (36). They claimed that people need regular interaction with others, and that this interaction must be positive and pleasant. Dysthe (37) identified teachers' good dialogue with their students as constituting good leadership in the teaching process. Such a dialogue will keep the students focused, engaged, and communicating.

Jordet (2) claimed that rights are a form of recognition, and that this recognition lies in the students experiencing themselves as active participants, i.e., that they are viewed as equals in the interaction. This implies that the teacher is friendly and listens to the student, which are indicators that the teacher respects the students and is working to ensure that they feel listened to and that their best interests are being focused upon. This form of recognition is close to the empirical finding of a good dialogue, both in this study and in Author (a) (7).

The third factor, feedback, was also identified in an interview study by Author (a) (7) as being important in being seen. Other research has reported similar findings. The questions in the category “feedback” correspond well with Federici and Skaalvik's (38) description of emotional and instrumental support. They addressed both instrumental support involving the experience of receiving advice, support and guidance, and the emotional aspect involving encouragement, appreciation, and a teacher who cares. Moreover, Cox et al. (6) asserted that students who feel supported by their teacher have a greater sense of belonging. Belonging is described by Deci and Ryan (14) as a fundamental need, which will therefore affect the experience of being seen. Theoretically, this significance can be explained in terms of the importance that people place on their resources and competencies being valued and recognised (2, 13), and which relates to being seen (7). To recognise involves giving praise and appreciation, and acknowledgement of what the person does or says (2). As the teacher is often a highly significant person in the students' lives, affirmative feedback from the teacher will help to fulfil these basic social needs, as claimed by Rosenberg (39). Author (b) (1) pointed out that the students had the feeling of being seen when the teacher saw their potential for achieving and further improvement by giving feedback. Gamlem (40) found that students prefer feedback not only related to what they do, but also what they can do better. The students in her study indicated the importance of such feedback in a good dialogue with the teacher, which supports the findings in the present study.

The fourth factor, being able to display one's skills, was also identified by Author (a) (7) in an interview study. A teacher who helps a student to see what he or she is good at, gives that student an opportunity to show what he or she is good at. Being able to display one's skills can be linked to the theory of recognition and social validation (2, 13), which is one of the three forms of recognition. This signifies that the individual's experiences of knowledge, qualities, and competencies are being valued. Feeling that one can contribute with one's abilities, and that these are valued, is a human need, according to Jordet (2). In school, this concerns the students experiencing varied activities that are adapted to their level of skill, so that they experience mastery and feel valued for their capabilities (2). The finding, i.e., being able to display one's skills, is supported by Engelsrud (41). She highlights that teachers have to be open-minded in their meetings with students, use open dialogue to know the students, and give the students the feeling that they are good enough as they are. With such an appreciative strategy, the teacher shows an acceptable, emphatic, and affirmative attitude in his or her meetings with students, independently of the students' abilities. Moreover, according to Lyngstad (42), such a strategy promotes professional and social learning, and develops self-esteem.

The fifth factor, evaluation and goals, is unique in that it was not identified by Author (a) (7) as a significant factor in being seen. Nonetheless, being included and treated fairly is in line with Honneth's (13) description of rights as a form of recognition. Jordet (2) pointed out that, from a school perspective, this refers to students' experience of being involved in active participation. This factor includes questions about goals and whether students are able to take part in setting goals for themselves. In addition, the factor includes questions about the evaluations that they receive from the teacher, and whether the students are permitted to take part in this goal setting and evaluation. To be given responsibility for one's own learning and assessment may well be experienced as a declaration of trust and, for the student, constitute a step towards self-realisation. The students getting to share in setting goals for themselves and in evaluating their own contribution is also consonant with autonomy as a fundamental need (14). Teachers show a belief in, and respect for, the students by including them in evaluation and goal setting, which can lead to the feeling that the teacher believes in and sees the student. As Author (b) (1) found in their study, the student feels seen when the teacher expresses respect and trust, and supports them in their learning process. Furthermore, the Norwegian Directorate of Education (43) pointed to the importance of including students in their own learning process, which is also related to evaluation and goals. In relation to evaluation and goals, they also point out that students should be able to evaluate their own progress, and reflect on their own development and learning. In other words, the students are the key to their own learning process, and the teachers have to involve them in their own learning process (44).

The results also showed that all five factors underlying the phenomenon of being seen correlated significantly with each other with moderate-to-high correlations (28). We contend that this can be explained by the fact that teachers' behaviour in one area (i.e., feedback) will affect the other four variables (i.e., the experience of a good dialogue in an evaluation in which the teachers care about the students).

Even if it is important that students experience being seen by their PE teacher, a study (1) found that being given much attention by the teacher could also be experienced negatively. This was the case with Marita (1). Marita reported that she was seen by her PE teacher, that this was positive, but she felt that she was seen too much, which created a negative feeling. She was in an education program in which most of her classmates were boys, and she had the feeling that the teacher needed to “mind her extra carefully” because she was a girl, and explained that she probably was seen too much because there were so few girls in the class. Marita stated that she felt that it was odd that the teacher needed to devote more attention to her and be especially considerate of her because she was a girl. She wanted to be treated on equal terms with the others.

### The correlation between factors underlying being seen and the experience of being seen

The results of the correlation analysis indicate that all five factors underlying being seen correlate significantly and positively at a medium-to-high level (28) with the experience of being seen. A high correlation (0.695) between the experience of the teacher caring and that of being seen suggests that it is important for the students that the teacher genuinely sees them and cares more for them as people. We have seen that a connection exists between the factors, and that they probably influence each other. We argue that being seen is closely associated with Jordet's (2) recognition theory, in which love as a form of recognition takes place through the teacher's caring, empathy, and warmth toward the students. The importance of the teacher's care is also emphasised in Jordet's (2) claim that love as a form of recognition is a fundamental condition for existence, and that it is a central part of the teacher's profession to exhibit love through empathy, care, and warmth. We assert that caring in PE is about letting the students show their skills, giving them feedback, having good dialogue with them, and involving them in evaluation and goal setting.

### Strengths and weaknesses of the study

In extant literature, there were no validated questionnaires or questions used to explore the phenomenon of *being seen*. For this reason, questions that had been validated for subjects closely related to students' experiences of the phenomenon of being seen were used, and some were developed in light of previous research (17, 18, 20, 21). The data are in accordance with Field's (24) considerations about the requisite size to be suitable for factor analysis, and the questions have a high face validity. This helped to strengthen the reliability and validity of this study. Furthermore, that four of the five factors identified as important for the experience of being seen in this quantitative study correspond with the findings of qualitative data (7) increases the reliability of the study. In addition, the Cronbach's alpha test showed that the internal consistency of the indexes is high (see Table 5). This indicates that the components of which the factors consist relate to each other, and thus we can conclude that the questions included in the five different indexes address the same issue. However, a larger sample from different parts of the country could still be beneficial in obtaining an even better representation of the country's upper secondary schools.

**Table 5:** Internal consistency of the factors measured with Cronbach's alpha test.

Factors	Cronbach's alpha
Feedback	0.939
Teacher's caring	0.946
Good dialogue	0.910
Evaluation and goals	0.871
Display one's skills	0.915

## Conclusion

This study examined the Norwegian pedagogical term *being seen*, which expresses what physical education (PE) teachers (and other teachers) say in everyday terms related to students' recognition, sense of belonging, and support by the teacher. This is the first study to identify factors that constitute the pedagogical term *being seen*, and uses a quantitative design. The results showed that 76.2% of the students experienced (to a certain extent) being seen by their PE teacher, while 7.8% (to a certain extent) experienced not being seen by their PE teacher. Even if there were few students who reported not being seen by their PE teacher, we argue that this is not satisfactory because of the teacher's unique possibility of playing a highly important role in the development of young people. Furthermore, the results showed that the pedagogical term *being seen* by the teacher in PE comprises five factors: (1) feeling that the teacher cares; (2) receiving feedback from the teacher; (3) having good dialogue; (4) engaging in evaluation and goal setting; and (5) having the experience of being able to display one's abilities. The correlation analysis showed that all five factors had a significant positive correlation with the experience of being seen, with moderate strength. Although the phenomenon of being seen has previously been studied only by means of qualitative interviews (1, 7), this study contributes new and worthwhile knowledge, capable of generalization to some similar subject contexts and educational contexts.. However, the unique nature of the subject of PE must carefully be reflected in this process.

Discussion of the results in relation to theory and earlier research suggests that being seen can be closely associated with Jordet's (2) recognition theory. Jordet (2) points to love as a form of recognition, and finds that this an important aspect of school life through the teacher's caring, empathy, and warmth. The findings are also related to Deci and Ryan's (14) theory about relatedness, as one of three basic needs in all humans. Further studies should examine being seen as a pedagogical term from the teachers' perspective, and also investigate what characterizes students who experience being seen and not being seen, especially related to gender and participation in organized sport. Future investigations should also strive to include more schools in their study for increased robustness and generalizability.

## Data Availability

The raw data supporting the conclusions of this article will be made available by the authors, without undue reservation.
